# Ubiquitination Insight from Spinal Muscular Atrophy—From Pathogenesis to Therapy: A Muscle Perspective

**DOI:** 10.3390/ijms25168800

**Published:** 2024-08-13

**Authors:** Alfonso Bolado-Carrancio, Olga Tapia, José C. Rodríguez-Rey

**Affiliations:** 1 Departamento de Biología Molecular, Facultad de Medicina, Universidad de Cantabria-and Instituto de Investigación Marqués de Valdecilla (IDIVAL), 39011 Santander, Spain; alfonso.bolado@unican.es; 2 Departamento de Ciencias Médicas Básicas, Instituto de Tecnologías Biomédicas, Universidad de la Laguna, 38200 La Laguna, Spain

**Keywords:** spinal muscular atrophy, ubiquitin–proteasome system, SMN, skeletal muscle atrophy

## Abstract

Spinal muscular atrophy (SMA) is one of the most frequent causes of death in childhood. The disease’s molecular basis is deletion or mutations in the *SMN1* gene, which produces reduced survival motor neuron protein (SMN) levels. As a result, there is spinal motor neuron degeneration and a large increase in muscle atrophy, in which the ubiquitin–proteasome system (UPS) plays a significant role. In humans, a paralogue of *SMN1*, *SMN2* encodes the truncated protein SMNΔ7. Structural differences between SMN and SMNΔ7 affect the interaction of the proteins with UPS and decrease the stability of the truncated protein. SMN loss affects the general ubiquitination process by lowering the levels of UBA1, one of the main enzymes in the ubiquitination process. We discuss how SMN loss affects both SMN stability and the general ubiquitination process, and how the proteins involved in ubiquitination could be used as future targets for SMA treatment.

## 1. Introduction

Striated skeletal muscle (SkM), the major tissue component of the body, contributes between 40 and 50% to its total weight in healthy adults. Beyond voluntary movement, SkM functions as the major storage for proteins and energy-rich compounds and interacts with major organs involved in metabolic processes through the secretion of soluble peptides or myokines [1,2]. Some myokines with autocrine/paracrine actions can also influence average myofiber growth [1]. Thus, muscle mass changes in response to several external and internal stimuli that influence the fine balance between muscle protein synthesis and breakdown. At the cellular level, stimuli might affect several signaling pathways and gene expression and influence muscle cells to adapt their mass and content accordingly. The stimuli that activate satellite cell proliferation and protein synthesis lead to myonuclear accretion and muscle hypertrophy [3,4]. In contrast, those deregulating the signaling pathways that induce myonuclear loss and protein degradation contribute to muscle atrophy.

Muscle fiber atrophy, a dramatic regression in myofiber volume, is the common response to both inherited and acquired pathologies that prevent normal contractile activity or pathological stimuli leading to increased protein breakdown. Some of such pathologies are congenital myopathies, inherited diseases characterized by the lack of muscle tone or floppiness at birth. Duchenne disease, congenital myotubular myopathy, or spinal muscular atrophy are representative of this group of inherited myopathies whose genetics and molecular mechanisms are well defined.

SMA is fundamentally a motor neuron disease. On this basis, most therapies aim to restore SMN levels in neurons. However, growing evidence shows that increasing SMN levels in skeletal muscle is also desirable (see Section 2 for a more detailed discussion). In this review, we focus on the role of ubiquitination in skeletal muscle cells and its change in SMA. A more detailed knowledge of this mechanism could bring to light new targets to raise SMN levels in muscles and, together with the therapies currently in use, contribute to the better management of SMA patients.

## 2. Spinal Muscular Atrophy (SMA)

**Figure 1:**
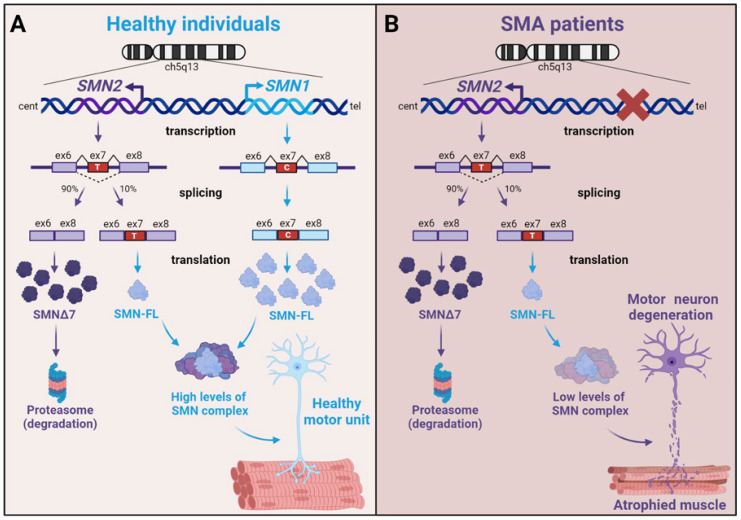
Generation of SMN transcripts from *SMN1* and *SMN2* genes. (**A**) In healthy individuals, *SMN1* and *SMN2* gene transcripts are translated into the full-length SMN (SMN-FL) and SMNΔ7 proteins, respectively. A small percentage of *SMN2* is also translated into SMN-FL. (**B**) In SMA patients, transcripts from *SMN1* are absent. Most *SMN2* transcripts are translated into SMNΔ7 and mostly degraded. See the text for a more detailed explanation. Figure created with BioRender.com.

Proximal spinal muscular atrophy (SMA) represents one of the most common genetic causes of infant mortality. SMA is an autosomal recessive neurodegenerative disorder characterized by a loss of motor neurons (α-MNs) from the anterior horn of the spinal cord. Muscle denervation leads to a neurogenic secondary myopathy, characterized by a progressive atrophy and weakness of the proximal limb and trunk muscles, which usually culminates in lethal respiratory failure. However, there is accumulated evidence that skeletal muscle in SMA is already affected in the pre-denervation phase. This is a devastating neuromuscular disorder caused by reduced levels of the survival motor neuron protein (SMN) [5,6]. In humans, *SMN* genes are two paralogues located as a large inverted duplication in chromosome region 5q13 resulting in *SMN1* (telomeric copy) and *SMN2* (centromeric copy) genes (Figure 1A). SMA typically results from the homozygous deletion of the *SMN1* gene, and the severity of SMA depends on the *SMN2* copy number. SMN genes are expressed ubiquitously in all eukaryotic cells and both *SMN1* and *SMN2* encode the full-length survival motor neuron protein (SMN). The *SMN1* gene produces high levels of transcripts encoded by nine exons (1, 2a, 2b, 3–8), of which exons 1–7 are translated into the full-length SMN protein (SMN-FL) [7,8]. In *SMN2*, a silent C-to-T transition within exon 7 favors an alternative splicing pattern generating high levels of an alternatively spliced isoform that lacks exon 7 (SMNΔ7) and low levels of SMN-FL transcripts (Figure 1B). These SMNΔ7 truncated transcripts are present in cells, but they encode a non-functional protein that is rapidly degraded by the proteasome and, therefore, barely undetectable [8,9]. The homozygous deletion or point mutations on the *SMN1* gene cause SMA, while the severity of the clinical outcome is directly directed by *SMN2* copy number [6].

**Figure 2:**
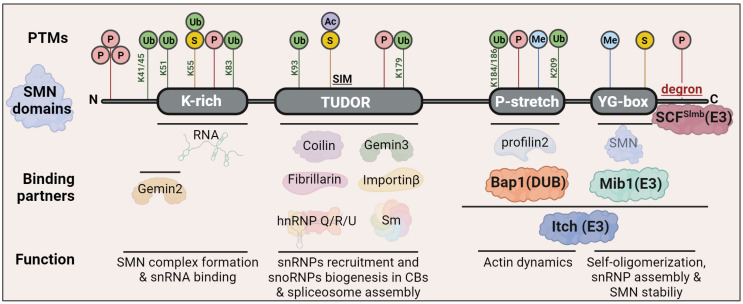
Diagrammatic representation of the domain structure of SMN-FL. The **SMN protein** comprises several highly conserved motifs: a basic lysine-rich domain (K-rich), a Tudor domain, a poly-L-proline-rich domain (P-stretch), and a Y/G box in close proximity to the C-terminus, that itself mediates self-oligomerization and stability. The SMN protein is highly modified through phosphorylation (P), methylation (Me), acetylation (Ac), SUMOylation (S), and ubiquitination (Ub). Depicted are a summary of sites modified by the indicated PTMs identified through MS/proteomics and other methods (see [19] for a complete list of SMN PTMs sites). Some of the well-known proteins that interact with SMN-FL and the corresponding function are depicted below the corresponding interacting domain (see [20] for a review on SMN interactors and functional implications). Also indicated are the known interaction sites of SMN with the E3 UBLs Mib1, Itch and SCF^Slmb^ and the DUB Bap1 (see Section 7 for a detailed discussion). Figure created with BioRender.com.

The SMN-FL protein is a multi-interacting protein of 294 amino acids that contains several highly conserved motifs (Figure 2). SMN-FL contains a basic/lysine-rich domain, encoded by exon 2, an interaction site with Gemin2 and RNAs [10], and Cajal body assembly [11]. Through the highly conserved Tudor domain, SMN interacts with the Sm proteins to facilitate the cytoplasmic assembly of spliceosomal ribonucleoproteins (snRNPs) [12]. It is also responsible for the interaction with p80-Coilin, the structural protein of Cajal bodies [13,14], Gemin5, an essential component of the SMN complex [15], or Fibrillarin [16], a nucleolar protein recruited into CBs for snoRNP biogenesis [17]. A polyproline stretch encoded by exon 4 is responsible for the binding of SMN with Profilin2a, an actin-binding protein implicated in the dynamic organization of the actin cytoskeleton [18]. Finally, the YG-box is required for SMN self-oligomerization [7]. Since monomeric SMN exposes a small degron within the YG box, SMN is found as a homopolymer [19] or complexed to Gemin2-8 and Unrip to form the SMN complex [9].

As part of the SMN complex, SMN has essential functions, with particular relevance to the cytoplasmic steps of spliceosomal snRNP biogenesis and their subsequent targeting of CBs before integration in the major and minor spliceosomes [21,22]. Since the splicing of pre-mRNA is an essential step in the maturation of mRNAs, the SMN-dependent reduction in snRNP biogenesis is considered a central pathogenic mechanism of SMA [23]. The SMN complex is also implicated in the anterograde transport of some mRNAs through the axon to the growth cone for their translation and in other relevant intracellular processes, such as actin cytoskeleton dynamics, ribosomal activity, endocytosis, mitochondrial homeostasis, or autophagy [24,25,26,27].

SMA has been historically classified as a motor neuron disease. Initial experiments carried out with transgenic mice showed that replacing SMN in neurons rescues the SMA phenotype and increases the animal’s survival whereas restoring SMN levels in muscle does not [28]. On this basis, most therapies are intended to replace SMN in motor neurons (see Section 7). However, there is increasing evidence that supports an independent contribution of the SkM to the pathogenesis of SMA [25]. The absence of the SMN protein causes intrinsic defects in myogenesis, with an altered expression of myogenic markers and myotube formation in both patient-derived type I SMA muscle cells and C2C12 murine myoblasts after siRNA-mediated SMN silencing [29,30]. In this vein, targeted knock-out experiments in mice have shown that reducing SMN in skeletal muscle results in a muscular dystrophic phenotype, necrosis, and altered neuromuscular junction, which finally leads to paralysis and eventually the death of the mice after 1 month of age [31,32] The restoration of SMN protein levels only at the CNS level partially rescues muscle cross-sectional area and myofiber diameter; however, the important structural damage of the sarcomere organization persists [33,34]. Furthermore, when SMA muscle cells are grown in the presence of healthy spinal cord explants, the degeneration of the co-culture is rapidly achieved, suggesting that muscle cells exert a retrograde neurotrophic effect essential for MN health [35,36,37]. Supporting this view is the fact that SkM from human SMA fetuses has a smaller size and is severely affected before MN degeneration [38]. These and many other data strongly support the view that muscles undergo independent pathological processes that contribute to the development of the characteristic neuromuscular dysfunction of SMA.

## 3. Ubiquitination, the Master Regulator of Protein Stability

**Figure 3:**
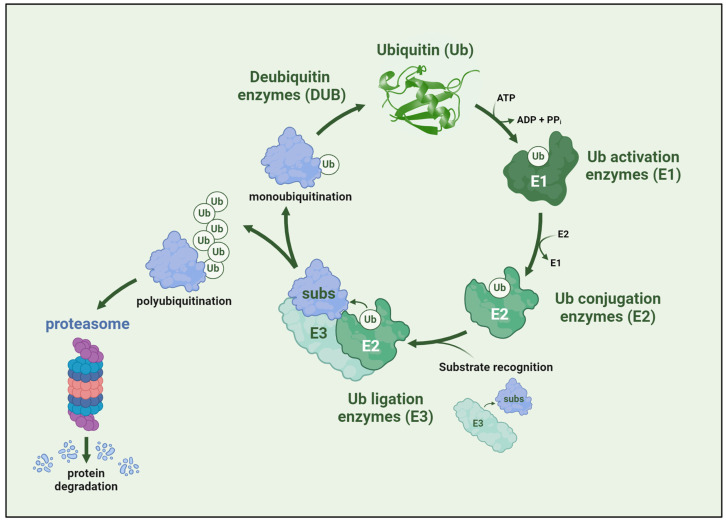
Outline of the ubiquitination process. An E1 ubiquitin-activating adenylates ubiquitin and forms an E1-Ub intermediate. Then, ubiquitin is transferred to the E2 ubiquitin-conjugating enzyme through a transthiolation reaction performed by E1. The E3 ligase forms an isopeptide bond between the substrate’s lysine side chain and the ubiquitin molecule’s C-terminal glycine. Monoubiquitinated molecules can either lose their ubiquitin moiety by the action of a deubiquitinase or become polyubiquitinated and further degraded in the proteasome. Figure created with BioRender.com.

At the very core of the mechanisms directly implicated in muscle protein degradation during muscle atrophy progression, regardless of the underlying cause, lies ubiquitination, the master regulator of protein turnover. Ubiquitination is a posttranslational modification essential for keeping cellular homeostasis and functionality. The process is typically defined as the covalent binding of the protein ubiquitin (Ub) to a target protein (Figure 3). Nonetheless, this process has been described in other biomolecules, such a lipids [39], although it is far less common than in proteins. Ubiquitin consists of 76 amino acids and is highly conserved among all eukaryotes; it was first described in 1975 [40] and identified as a conjugated protein in 1977 [41]. Since then, other proteins have been identified to have a similar function and structure as ubiquitin, forming the ubiquitin-like protein family [42]. Nevertheless, ubiquitin seems to be the most relevant member of the family, as the ubiquitin–proteasome system mediates the large majority of protein degradation.

Although the regulation of protein stability is the most notable and well-studied role of ubiquitin, it is not the only one; different reports showed that ubiquitin can also affect protein activity, localization, DNA damage response, or cell cycle progression, a list of roles that keep growing and highlights the relevance and versatility of this PTM [39,43,44,45]. Differently from other UBLs, ubiquitin can make heterotopic or homotypic polyubiquitin chains through the covalent binding to one of seven different lysine residues (K6, K11, K27, K29, K33, K48, and K63) and the N-terminal Methionine [43,44]. Of these, the two most studied are the “canonical” K48-linkages, which target substrates for proteasomal degradation, and K63-linkages associated with vesicular trafficking, autophagy, and the modulation of the NFKB pathway [39,43,44]. The other five types are considered less abundant or atypical and are still poorly understood [44,45,46].

Ubiquitination is a reversible and sequential process that requires three different ligases that take the ubiquitin until it is transferred to the substrate [47] (Figure 3). Ubiquitin is adenylated (activated) in the C-terminal glycine by an E1 ubiquitin-activating enzyme, forming an E1-Ub intermediate via a thioester bond. The ubiquitin is then transferred to the E2 ubiquitin-conjugating enzyme through a transthiolation reaction performed by the E1 ligase [48]. The E3 ligase transfers the ubiquitin from the E2 ligase to a lysine side chain of the substrate via an isopeptide bond with the C-terminal glycine of the ubiquitin [49,50]. Although non-lysine ubiquitylation has been described, it seems far less abundant than canonical lysine-ubiquitylation [44,51]. During the ubiquitylation process, E3 are the ligases that interact with the substrate. Due to this, they are the ones that provide specificity to the ubiquitination process through the recognition of specific motifs denominated as degrons [49]. Consequently, E3 ligases are far more abundant than E1 and E2 ligases, with over 600 genes coding for E3 ligases. In comparison, there are only two canonical E1 ligases for ubiquitin (UBA1 and UBA6) and around 40 E2 ligases [52]. Because of their more specific nature, researchers have hypothesized about the therapeutic potential of inhibiting E3 ligases in different diseases, such as cancer [53], metabolic diseases [54], or muscular atrophy [55].

Based on their structure and mechanism of action, E3 ligases can be divided into three families: HECT, RING-finger, and RBR. Nonetheless, classification varies as other authors classify them into two types based exclusively on the domain responsible for the E2-E3 ligase interaction. In this case, the families would be HECT and the RING-finger superfamily, to which the RBR would belong [56].

HECT was the first family of E3 ligases described [57]. The members of this family are characterized by the shared catalytic HECT (Homologous to the E6-AP Carboxyl Terminus) domain, which gives its name to the family. The HECT domain is located in all characterized ligases at the C-terminal [58]. It expands for around 350aa and is subdivided into a C-lobe and an N-lobe. As to their mechanism of action, forming a thioester bond between the E3 ligase and the ubiquitin always precedes the transfer of the ubiquitin to the substrate. This generates an E3 ligase–ubiquitin intermediate. The N-lobe of the HECT domain is responsible for the interaction with E2 ligase, and the C-lobe contains active cysteine that helps establish the thioester bond with ubiquitin [59].

RING-finger E3 ligases comprise most E3 ligases, with around 90% of the known E3 belonging to this family [60]. The members of this family are characterized by the presence of a RING (Really Interesting New Gene) domain. This domain coordinates the zinc ions, the key to the E2-E3 ligase interaction [61]. The members of this family do not form a thioester intermediate; instead, the E3 ligase works as a scaffold, facilitating the ubiquitin transfer to the substrate, but without an E3-Ub intermediate. Although some RING ligases are monomeric, many can form dimers or multi-subunit complexes. Among dimers, homodimers can interact with two different E2 ligases simultaneously [62], while in most heterodimers, one E3 ligase serves as “true” E3 ligase, while the other enhances the activity or the stability of the E2-E3–substrate complex [62] as is the case for BRCA1-BARD1 [63]. Oligomer formation is not an exclusive characteristic of RING ligases, and in HECT E3 ligases, it is an important activity modulator [62]. Sometimes, as in E6AP, oligomerization is an activation mechanism, but in other cases, as with the MyoD regulator HUWE1 [64], homodimer formation represses its activity [65]. The multimeric E3 ligases comprise several subunits, where the E2 ligase and the substrate can interact with different ones [50]. One notable type of multimeric RING E3 ligases is cullin-RING (CRLs). CRLs are characterized by the presence of subunits with an F-box domain, which is responsible for the substrate specificity, that interacts with the N-terminus of the central cullin, whereas the C-terminus interacts with an E3 RING ligase [66]. Some of the ligases of this family have a U-Box domain that adopts the same conformation and function as a RING domain, but it cannot coordinate Zinc due to the lack of cysteines [67]. The two most-studied E3 ligases in muscle belong to this family, the pro-atrophy E3 ligases MuRF1/TRIM63 and Atrogin-1/FBXO32.

The third E3 ligase family, RBR (RING-between-RING), stands between the HECT and RING E3 ligases. The members of this family have a RING1, an in-between RING (IBR) and a RING2 domain. The RING1 domain functions similarly to the RING domain of a RING E3 ligase, binding the UB-E2 ligase [68], and it has the same 3D structure. The difference with RING E3 ligases is that the ubiquitin is then transferred to a cysteine in the RING2, forming a thioester bond similar to HECT E3 ligases [68,69]. The IBR domain would serve as a docking site for ubiquitin necessary for the allosteric activation and feed-forward mechanism that suppresses the autoinhibitory state [53].

## 4. Role of E3 Ligases in Muscle Atrophy Development

### 4.1. MuRF1 and Atrogin-1

The two main E3 ligases associated with atrophy are the RING E3 ligases MuRF1/TRIM63 and Atrogin-1/MAFbx [70]. Since their discovery in 2001, they have become the most studied E3 ligases in muscle atrophy and are upregulated in most atrophies, including SMA [30]. FoxOs induce both MuRF1 and Atrogin-1, although some differences in the ability of different FoxOs to regulate their expression have been found [71,72,73]. FoxOs are translocated to the nucleus and upregulate their expression [71,72]. Other pathways promote or synergize with FoxO; these include Wnt/beta-catenin [74], AMPK [75], TNF-α [76], SMAD2/3 [77,78], Glucocorticoid Receptor [79], p38 [32,80], JAK/STAT3 [81], C/EBPdelta and ATF4 [82], or Notch2 [83]. NF-KB induces atrophy and MuRF-1 expression but not Atrogin-1 [84,85].

MuRF1 regulates the degradation of several essential muscle proteins. MuRF1 overexpression led to the identification of 56 potential ubiquitination substrates, including Creatine Kinase, p62, or the myosin regulatory light chains MyLC1 and MyLC2 [86]. MuRF1 induces the degradation of actin [87] and Myosin heavy chain (MYH) in dexamethasone-treated mice [88] and C2C12 myotubes [89]. It regulates the degradation of cardiac troponin-1 [90] and interacts with titin, disrupting M-line assembly [91]. In addition, MURF1-KO mice show metabolic alterations similar to those in Diabetes Mellitus Type 2 [92]. Overall, these targets highlight the role of MuRF1 in promoting atrophy and a loss of muscular contractility. Nonetheless, it has been recently shown that MuRF1 promotes the replacement of myosin-heavy chains 3 and 4 in a ubiquitination-independent manner [93], suggesting a role in thick filament myosin replacement.

In contrast, Atrogin-1 targets are more eclectic. In myostatin-treated C2C12 myotubes, Vimentin, Desmin, and the Myogenic Regulatory Factor MyoD were identified as substrates of Atrogin-1, among other 78 potential substrates [94]. The eukaryotic initiation factor 3 subunit 5 (eIF3-f), associated with hypertrophy induction, was also identified as a substrate of Atrogin-1 [95]. In an injury model, atrogin-1 targets aquaporin-4 for degradation, which might be involved in myocyte shrinkage [96]. Nevertheless, atrogin-1 has been described as having some protective roles. In cardiomyocytes, it promotes calcineurin A degradation, [90] protecting against cardiac hypertrophy; and in a zebrafish model of Duchenne, the upregulation of atrogin-1 muscular function via the degradation of the endoplasmic reticulum chaperone BiP was observed [97]. These findings suggest that in opposition to MuRF1, Atrogin-1 has some role in muscle mass protection in some pathological processes. The observed upregulation of MuRF1 and Atrogin-1 in SMA [30] led to the hypothesis that reducing their levels would ameliorate the disease progression. While some potential treatments focus in the reduction for both atrogenes, such as the Growth hormone-releasing hormone (GHRH) and a synthetic analog [98], others have found that the modification of MuRF1 and Atrogin-1 alone plays no role in disease progression in the SMNΔ7 SMA mouse [99]. This suggests that it is likely that the alterations found in ubiquitin homeostasis in SMA are more profound and have more players. Therefore, it is worth discussing the other E3 ligases associated with muscle atrophy.

### 4.2. E3 Ligases Associated with MuRF-1 and Atrogin-1

Recent studies have found other E3 ligases that are upregulated with MuRF-1 and Atrogin-1 to promote atrophy, which could be of interest in SMA given the fact that the inhibition of MuRF1 and Atrogin-1 does not rescue the SMA phenotype in the SMNΔ7 SMA mouse [99]. SMART and MUSA1 are two E3 ligases whose expression is SMAD and FOXO dependent in muscle and are expressed together with Murf-1 and Atrogin-1 in skeletal muscle atrophies [100,101,102]. Ube4a is an E3 ligase associated with FOXO induction of mitochondrial decline in aging [103]. FBLX22 is an atrophy-promoting E3 ligase in an additive mechanism to MuRF1 [104]. Another E3 ligase associated with this is Trip12, an HECT E3 ligase that regulates the switch from slow to fast fibers via the degradation of Sox6 in C2c12 myotubes [105]. The fiber-type switching process has been associated with the induction of muscle atrophy [106] and suggests that Trip12 could be part of coordinated muscle atrophy. In line with this, treatment with Isoquercitrin, an Nrf2 agonist [107], inhibited the slow-to-fast fiber type conversion and atrophy induction [108], suggesting that Nrf2 is a therapeutic target for muscle wasting [109]. As Nrf2 is downregulated in SMA [110], this opens the question about the potential of Nrf2 upregulation in SMA to ameliorate disease progression, as well as fiber-switching role in SMA.

### 4.3. PI3K/AKT and Other Guardians of Muscle Mass

The activation of the AKT/PI3K plays a dominant role in protein synthesis and atrophy protection (reviewed in [111]); therefore, it is not surprising that PI3K/AKT activity is altered in several SMA [112,113]. PI3K/AKT downregulation has been associated with reduced SMN protein [112], possibly due to a hyperactivation of the MAPK/ERK pathway [113]. In line with this, ERK inhibition induces *SMN2* expression via the PI3K/AKT pathway [114]. The dysregulation of the putative regulator of PI3K/AKT pathway hsa-miR-663a [115] reinforces the notion that alterations in PI3K/AKT pathway are a common feature in SMA and that the classic interplay between PI3K/AKT-FoxO-B-catenin found in atrophy, might be of capital relevance in SMA.

The downregulation of PI3K/AKT is a common theme in other muscle atrophies. PI3K/AKT downregulation by the E3 ligase Trim32 induces muscle atrophy via the disruption of the plakoglobin–p85 interaction [116], as well as targeting the degradation of several myofibers components (reviewed in [117]). Trim32 mutations are associated with the myopathy Limb Girdle Muscular Dystrophy or LGMD2H [118,119,120] and induce the degradation of thin filaments and the z-band [121]. Interestingly, Trim32 is not induced in classic models of FOXO-associated atrophy, with ref. [121] suggesting that the Trim32 of regulation is FOXO-independent. Conversely, Trim32 induces autophagy during fasting through the ubiquitination of ULK1 [122]. As dysregulated autophagy levels do not have a protective role against atrophy [118], this might imply that Trm32 is a protective mechanism against fasting-induced atrophy. Interestingly, Trim32 is a substrate for autophagy via p62, while the mutants associated with LGMD2H are not [123]. The monoubiquitylation of p62 by Trim32 suggests that the alteration in p62-mediated degradation could contribute to pathology. This and the independence from the classic atrophy induction suggest that Trim32 plays a much more complex role in muscle mass regulation than its associated disorders suggested.

An E3 ligase that potentially regulates PI3K/AKT in muscle is the CRL1 E3 ligase Fbxo31. Fbxo31 is an atrophy-associated E3 ligase that is also upregulated by FOXOs in a non-direct manner [100]. FBXO31 expression has been associated with the downregulation of the PI3K/AKT and MAPK/ERK pathways in prostate cancer [124] through DUSP6 degradation, a key regulator of MAPK/ERK-mediated growth in muscle [125]. Interestingly, AKT promotes Fbxo31 degradation in HEK293 cells [126], while in cervical cancer cells, Fbxo31 inhibits the AKT-MDM2 axis [127]. As MDM2 targets FOXOs for degradation in muscle [128], this opens a possibility where Fbxo1 activity can be a cross-regulatory node for AKT and FoxO to maintain muscle mass. On the other hand, supporting a pro-atrophic role, Fbxo31 is upregulated in glucocorticoid-induced atrophy [129] and promotes SMAD7 degradation in liver cancer [130]. SMAD7 plays a protective role against myostatin-mediated atrophy in skeletal muscle [131], in opposition to SMAD2/3 [78]. In agreement with this, using myostatin inhibitors, an important SMAD2/3 activator in muscle, is a promising therapeutic approach in SMA [132,133]. This could suggest that increased FoxO/B-catenin activity could lead to increased Fbxo31 levels, which would degrade SMAD7 and downregulate PI3K/AKT, preventing the inhibition of the atrophy expression genes. All of these argue for the study of Fbxo31 in the coordination of signaling pathways regulating muscle mass and atrophy in SMA and other muscular atrophies.

Recently, a mouse model with defects in BMP binding with the receptor MuSK (muscle-specific kinase) displayed muscle mass reduction with a dysfunctional PI3K/AKT pathway [130], which highlights the interplay between both pathways in maintaining and promoting muscle mass. BMP is a relevant pathway in muscle development [134] that promotes hypertrophy and protects against atrophy via the downregulation of MUSA-1 [135]. Indeed, in cancer models, the expression of the BMP inhibitor noggin promotes cachexia [136], indicating the relevance of BMP as a muscle mass maintenance pathway in stress conditions. In addition, BMP agonists have been suggested to be of interest, as in a drosophila model of SMA, they help maintain the neuromuscular junction integrity [137].

Although the ubiquitination and upregulation of specific E3 ligases play a significant role in muscle protein degradation and atrophy progression, they can also protect against atrophy and promote cellular and tissue recovery and even hypertrophy. Such is the case of Ubr5, an HECT E3 ligase implicated in muscle hypertrophy and recovery from atrophy [138] and the activity of the PI3K/AKT pathway in cancer [139,140]. Ubr5 knock-down in mouse models induces atrophy with a reduction in ERK and AKT activity and a chronic increase in S6K1 phosphorylation [141]. S6K1 hyperphosphorylation has been previously shown to induce MuRF1 and atrogin-1 in mice [142]. In line with this, the Ubr5 expression pattern alternated with the observed for MuRF1 and Atrogin-1 [138]. This makes the modulation of Ubr5 expression and/or activity of interest in SMA as it can be a compensatory mechanism for the observed dysregulation of ERK and AKT in SMA models.

## 5. FOXOs as Regulators of Muscle Mass

IGF-PI3K-AKT is the major anabolic pathway in skeletal muscle. Among other effects, activated AKT unblocks the translation initiation factor eIF2 through GSK-3β phosphorylation. Also, the activation of the mTORC1 complex by AKT results in the activation of both p70S6K and eIF4E (see Chen et al. for a Review [72]). On the other hand, muscle catabolism is the response to different types of stress (metabolic, inflammatory, and oxidative), where transcription factors of the FOXO family regulate the cell adaptation to stress.

The FOXO family comprises four members: FOXO1, 3, 4, and 6, all expressed in skeletal muscle [143]. Some specific functions have been attributed to a particular isoform [144], but in terms of regulation, FOXO1, 3, and 4 are very similar and, when activated, all induce muscle atrophy. On the contrary, FOXO6 seems to protect muscle from atrophy and also shows differences with the other isoforms in regulation [145]. Several pathways induced by different types of stressors converge and activate FOXOs, which elicit two proteolytic systems involved in muscle atrophy: autophagy [146] and UPS [147]. As mentioned before, FOXO 1, 3, and 4 regulate the expression of several atrophy genes [148], including MuRF1 and Atrogin-1 [71,72,73]. Posttranslational modifications, including phosphorylation, acetylation, and ubiquitination, are major mechanisms of the regulation of FOXO activity (see Rodríguez-Colman et al. for a recent review on FOXO regulation [147]). FOXO has several phosphorylation sites, the phosphorylation of which often results in contrary effects. For example, phosphorylation by AKT at serine 256 allows FOXO1 binding to 14-3-3 protein, masks the nuclear localization signal, and results in the shuttling of FOXO to the cytoplasm. On the contrary, AMPK phosphorylation favors the nuclear location of FOXOs and increases their transcriptional activity.

### 5.1. The Interplay between FOXO and Ubiquitination

Ubiquitination also plays different roles in FOXO regulation. Minute double minute 2 (MDM2) promotes the ubiquitination of FOXO1, 3, and 4 and is regarded as the general E3 ligase for FOXO protein degradation [128]. Under oxidative stress conditions, MDM2 monoubiquitinated FOXO. Monoubiquitylation can target proteins for degradation, but the affinity of monoubiquitinated proteins for the 26S proteasome is weak, and proteasome preferentially degrades proteins with at least four ubiquitin residues [149]. Consequently, the monoubiquitylation of FOXO does not result in the degradation of the protein, but it promotes nuclear location and increases FOXO transcriptional activity instead [128,147]. The process can be reversed by deubiquitylation by USP7/HAUSP, a FOXO6-induced deubiquitinase [150] that also protects MDM2 against degradation [150]. Also, especially when in high levels, MDM2 can also induce the polyubiquitination of FOXOs. Moreover, the polyubiquitination of FOXOs is also induced by the branching E3 ligase SKP2 [128,147].

The complex posttranslational regulation of FOXOs ensures the plasticity that allows them to respond adequately to different stress signals [128,151]. This plasticity is increased by the ability of FOXOs to act in synergy with factors induced by other signaling pathways. For instance, inflammation-induced NFκB and SMAD act synergistically with FOXO to elicit the expression of atrogenes [72]. FOXOs bind transcriptional regulators such as acetyl transferases (p300/CBP, PCAF) and deacetylases like SIRT1 and HDC6 to regulate gene transcription. The binding of these enzymes has a double effect on FOXO transcription. On the one hand, they promote chromatin remodeling, thus enabling the FOXO activation of the transcription, but they also regulate the acetylation of lysines within the DNA binding region of FOXO. This, in turn, would alter the DNA affinity of FOXO and impair its transcription activity [147]. β -catenin, initially identified as a binding partner of FOXO3 [152], is known for its ability to potentiate the recruitment of co-regulators [153], and the binding of FOXO4 to β-catenin helps to recruit acetyl-transferases like CBP/P300. The binding regions of FOXO to β-catenin have been recently mapped, and the binding would relieve the state of auto-inhibition of the CBP/P300—FOXO complex caused by the acetylation of FOXO [154]. β-catenin activity has become of interest in the SMA field due to the pharmacological inhibition of β-catenin as a potential therapeutic approach for SMA [155]. Phosphorylation by AKT changes the conformation of the β-catenin-binding region of FOXO4, impairs the binding, and might contribute to FOXO exclusion from the nucleus. The conservation of binding areas among all the FOXOs suggests that this mechanism could be shared by all of them rather than being exclusive to FOXO4 [154].

### 5.2. β-Catenin and AMPK: Partners of FoxO in Atrophy

B-catenin is a pro-atrophic pathway [156] that synergizes with FoxO1 [157]. Canonical Beta-catenin has been widely associated with the physiopathology of SMA [158] and other neuromuscular diseases, such as Spinocerebellar ataxia [157], where the mutation of the gene encoding for the E3 ligase CHIP is the underlying cause. Consistent with this, CHIP KO models show age-related muscle loss [159]. However, no data relates β-catenin to CHIP in muscle. The contribution of β-catenin to SMA will be discussed in more detail below. Like β-catenin, AMPK activation, specifically AMPKα2, is an inductor of atrophy via FoxO3 and autophagy activation [160,161]. In response to glucose levels, AMPKα2 is targeted for degradation in C2C12 myotubes by the E3 MG53 [162]. MG53 also promotes mitophagy through AMBRA1 [163]. The accumulation of dysfunctional mitochondria in the muscle is characteristic of SMA [164,165], and the upregulation of this process might be of interest. On the other hand, AMPK is essential for muscle mitophagy [160]. In the SMNΔ7 mouse model, treatment with the AMPK agonist AICAR improved skeletal muscle function but did not prevent motor neuron dysfunction and death [166]. Mitochondrial dysfunction can, in turn, induce an AMPK-mediated promotion of muscle atrophy [167]. These data suggest that correct mitophagy function in SMA outweighs the AMPK-mediated FoxO3 activation, highlighting the need to study how to boost mitophagy in SMA.

Results of studies on PARKIN expression levels suggest that PARKIN promotes muscle regeneration and protects against atrophy via mitophagy and protection against muscle wasting during fasting [168,169,170,171]. In non-muscle cellular models, the AMPK-dependent activation of PARKIN increases mitophagy via ULK-1, making PARKIN an attractive therapy target [172].

## 6. Changes in Ubiquitination in SMA

Numerous factors belonging to the ubiquitin–proteasome system have been shown to regulate the stability and degradation of both SMN-FL and SMNΔ7 [173,174]. SMN has about twice the half-life of the truncated variant SMNΔ7 [175], which can be explained by changes in the ubiquitination process of each protein. Thus, whereas SMN is mostly monoubiquitinated, SMNΔ7 is heavily polyubiquitinated, which likely promotes rapid proteasome degradation and would explain its short half-life [176].

Several E3 Ub-ligases have been reported as directly responsible for SMN mono- or polyubiquitination. Mib1 is the most well-known E3 ligase that ubiquitinates SMN among them. The N-terminal domain of Mib1 and the segment of SMN encoded by exon 6 of SMN are the regions responsible for this interaction. The overexpression of Mib resulted in a greater ubiquitination of SMNΔ7 compared to SMN-FL while knocking down Mib1 increased the stability of both SMN-FL and SMNΔ7 proteins [177,178]. Another E3 ligase that operates in SMN ubiquitination is UCHL1. Firstly identified as a member of the ubiquitin carboxy-terminal hydrolase family of deubiquitinating enzymes, UCHL1 ubiquitinates SMN by acting as an ATP-independent ubiquitin ligase [179]. A proteomic analysis of skin fibroblasts of SMA patients showed increased amounts of UCHL1, and treatment with purified UCHL1 induced the ubiquitination of SMN in vitro [180]. However, the inhibition of UCHL1 failed to improve SMA mice’s survival and resulted in increased weight loss. A plausible interpretation of these data is that UCHL1 increases as compensation for the decrease in UBA1, which is seen in SMA [181] (see below).

Another E3 SMN Ub-ligase acting on SMN is Itch. The silencing of Itch results in an SMN increased half-life, suggesting that ubiquitination by Itch may also have a role in SMN degradation [182]. Ubiquitination by Itch and the subsequent degradation of SMN is accelerated after the SUMOylation of SMN [183]. However, the role of Itch in the regulation of muscle mass could be more complex. The overexpression of Itch in cardiac muscle protects against cardiac hypertrophy via the inhibition of the Wnt/Beta-catenin pathway [184]. In line with this, a patient with a loss of Itch showed increased non-aging-related muscle wasting and mitochondrial dysfunction [185]. As to the control of SMN stability, Itch interacts directly with both SMN and SMNΔ7. As a result, SMN and SMNΔ7 become monoubiquitinated. Although mono-ubiquitination is often an insufficient degradation signal, it might act as a primer for further ubiquitination and SMN degradation. Itch-mediated ubiquitination might also regulate the correct intracellular distribution of SMN and, thus, the integrity of Cajal bodies and snRNP maturation [182].

**Figure 4:**
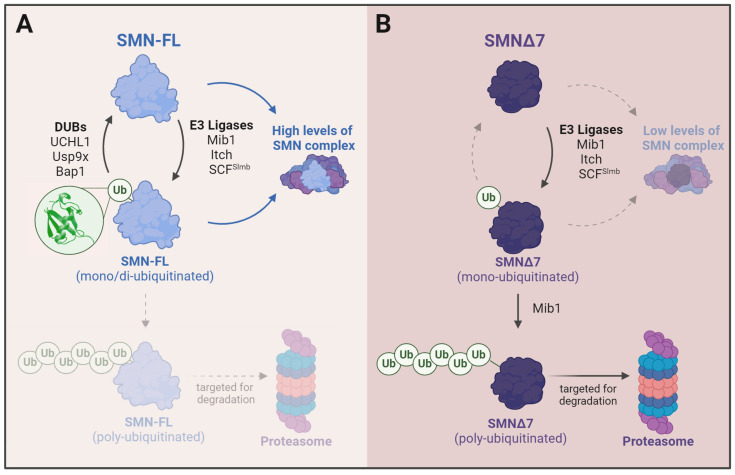
(**A**) Mono-ubiquitination of SMN would not preclude its incorporation into the SMN complex. The incorporation of SMN into the complex, in turn, would prevent SMN from polyubiquitination and proteasomal degradation. Mono-ubiquitination can be reversed by Usp9x and Bap1. (**B**) The inability of SMNΔ7 to form stable complexes, its decreased affinity for Usp9x and its different compartment distribution would make SMNΔ7 more prone to polyubiquitination and degradation by the UPS. Figure created with Biorender.com.

The fact that SMN protein is mostly monoubiquitinated allows another form of regulation based on the activity of deubiquitinases [186]. Three enzymes able to deubiquitinate SMN have been described so far: UCHL1, the role of which remains controversial; UCHL2 (also known as Bap1), another member of the ubiquitin carboxy-terminal hydrolase family [187]; and Usp9x. Usp9x is a protein that deubiquitinates mono- and di-ubiquitinated SMN. The knock-down of Usp9x in mammalian cells promotes SMN degradation and reduces SMN protein levels. However, Usp9x does not deubiquitinate nuclear SMNΔ7. The different conformation of SMNΔ7 could likely alter the affinity for Usp9x. Also, SMNΔ7 is located preferentially within the nucleus, making it less accessible to the mostly cytoplasmic Usp9x [176]. A plausible model of SMN regulation by ubiquitination is shown in Figure 4. The mono-ubiquitination and di-ubiquitination of SMN would not preclude its incorporation into the SMN complex and could be reversed by Usp9x and Bap1. The incorporation of SMN into the complex, in turn, would prevent SMN from polyubiquitination. The inability of SMNΔ7 to form stable complexes, its decreased affinity for Usp9x, and its different compartment distribution would make SMNΔ7 more prone to polyubiquitination and degradation by the UPS.

Changes that affect SMN stability are not the sole differences in ubiquitination in SMA pathology. Wishart et al. showed a reduction of about 60% in the levels of UBA1, an E1 ubiquitin-activating enzyme, in the muscle of murine models of SMA [155]. The role of UBA1 in the pathology of SMA is further supported by the identification of mutations in UBE1, the UBA1 coding gene protein in SMA-like pathologies such as an X-linked infantile form of SMA, and spinal and bulbar muscular atrophy (SBMA) [188,189,190]. The restoration of UBA1 systemic levels through genetic manipulation decreases SMA severity [191].

**Figure 5:**
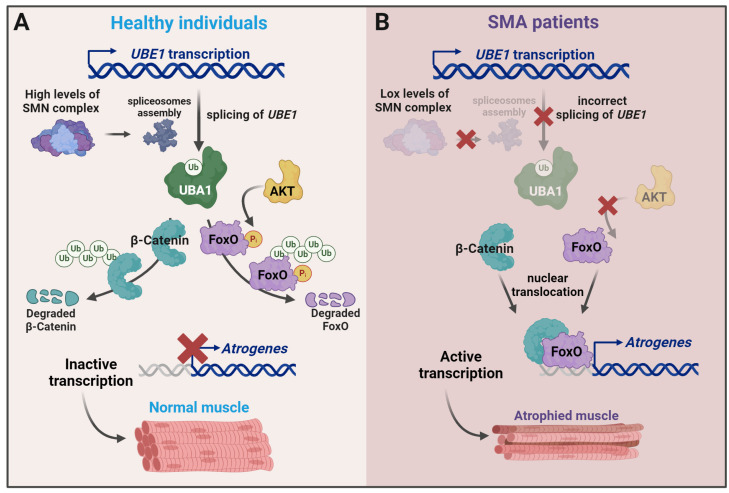
A model to explain the functional link between SMN levels and ubiquitination and the changes in SMA. (**A**) Functional SMN complexes and correct UBE1 splicing render normal levels of UBA1 protein, which drive the ubiquitination and degradation of β-catenin and block the transcription of atrogenes. AKT activation reinforces the effect by phosphorylating FOXO and making it more sensitive to ubiquitination by MDM2. (**B**) In SMA patients, reduced levels of SMN result in low levels of UBA1, the accumulation of undegraded β-catenin, and increased atrogene expression. Figure created with Biorender.com.

UBA1 is an E1 ubiquitin-activating enzyme, and a reduction in UBA1 levels generally alters the ubiquitination process. One of the consequences of the decrease in UBA1 levels is the accumulation of β-catenin, which in turn can interact with FoxO1 to promote its atrophy program [74]. The abnormal regulation of WβC signaling is involved in the pathogenesis of several neurodegenerative diseases, including SMA [157,158], and the activation of β-catenin pathway in skeletal muscle mesenchymal progenitors leads to muscle mass loss [156]. The effect of the treatment with quercetin, a specific inhibitor of β-catenin, highlights the role of β-catenin signaling. Quercetin reverses the impact of UBA1 loss in neurons and skeletal muscle, suggesting that, at least in these two tissues, β-catenin constitutes a functional link between UBA1 and neuromuscular loss [155]. Uba1 splice variants, Uba1a and Uba1b, are generated from the Ube1 gene. The absence of SMN alters the Uba1a to Uba1b ratio and the cellular distribution of the splicing isoforms in the spinal cord of Taiwanese SMA mice. The disruption of the splicing correlates with the reduction in UBA1 protein levels [155]. Thus, the known role of SMN in splicing regulation could explain the decrease in UBA1 levels observed in SMA (Figure 5).

## 7. UPS-Directed Approaches in SMA Therapeutics

Over the past decade, significant progress has been made in the treatment of SMA and the restoration of SMN levels is the primary goal of SMA therapy. Current SMA treatment options in clinical use, Nusinersen, Onasemnogen abeparvovec, or Risdiplam, are gene therapies directed to increase SMN protein levels by enhancing the inclusion of exon 7 into *SMN2* mRNA transcripts with RNA splicing modulators or by means of *SMN1* gene-targeting delivery using an adeno-associated virus 9 vector [192]. However, full-length SMN protein levels can be enhanced by acting on those posttranslational modifications that increase SMN protein stability. Salbutamol was a drug primarily used to relieve symptoms of asthma and chronic obstructive pulmonary disease and is now part of the SMA therapeutic arsenal [193]. As a β2-adrenergic receptor agonist, salbutamol induces PKA signaling. Phosphorylation by PKA SMN favors SMN complex formation, stabilizing SMN [194]. Zolgensma is another potential gene-replacement therapy; however, clinical trial data reduce the expectations. Recently, it has been proposed that zolgensma treatment in conjunction with the expression of the ubiquitination-resistant variant of survival motor neuron (SMN), SMNK^186R^, might be a good approach [195].

Researchers are constantly exploring how to exploit the ubiquitination system to their advantage for targeted protein degradation, the most notorious case being proteolysis-targeting chimera technology or PROTAC [196]. The actual focus is on strategies directed to interfere with the E3 enzymatic mechanism, inhibit the expression of deubiquitinases (DUBs), the family of enzymes responsible for the removal of conjugated ubiquitin, or modulate those signaling pathways responsible for E3 or DUB expression and/or activation.

Due to its essential role in SMA, the UBA1–catenin axis offers many therapeutic possibilities. The systemic restoration of UBA1 using an adenovirus increased survival and motor ability in SMA mice [191]. Auranofin, a small molecule already approved for treating rheumatoid arthritis, enhances UBA1 interaction with several E2 ubiquitin-conjugating enzymes and activates downstream E3 ligases [197]. Quercetin, a polyphenolic flavonoid that acts as a β-catenin inhibitor, also ameliorates neuromuscular pathology in several SMA animal models [155]. Quercetin’s clinical use is restricted by its low bioavailability, but new pharmaceutical formulations are being developed to circumvent this inconvenience [198]. In summary, current treatments for SMA do not appear to be effective in all patients, and there is a need for drugs that do more than just restore SMN levels [199]. Since the loss of SMN leads to huge alterations in the ubiquitination process, molecules involved in these processes could provide new targets for treating SMA.
